# Unexpected Hepatic Uptake of Tc-99m-MAA in Lung Perfusion Scintigraphy in a Patient with End-stage Renal Disease

**DOI:** 10.4274/mirt.galenos.2018.55264

**Published:** 2019-03-19

**Authors:** Kadir Alper Küçüker, İsa Burak Güney, Kairgeldy Aikimbaev, Saime Paydaş

**Affiliations:** 1 Çukurova University Faculty of Medicine, Department of Nuclear Medicine, Adana, Turkey; 2 Çukurova University Faculty of Medicine, Department of Radiology, Adana, Turkey; 3 Çukurova University Faculty of Medicine, Department of Internal Medicine, Adana, Turkey

**Keywords:** Tc-99m-macroaggregated albumin, perfusion scintigraphy, collateral circulation, liver, end-stage renal disease

## Abstract

Extra-pulmonary accumulation of Tc-99m-macroaggregated albumin (MAA) is described as uptake areas out of the lung in perfusion scintigraphy. If the particles spread throughout the body before reaching the lung via venous collaterals or due to right-to-left shunt, or if the particles are too small to occlude the pulmonary capillaries, then the agent can be seen at different locations of the body. Extra-pulmonary accumulation of Tc-99m-MAA can be detected mostly in the liver as well as in the brain, kidney, thyroid, myocardium, spleen and vertebra. Herein, we present lung scanning images with unexpected hepatic accumulation of Tc-99m-MAA. This pulmonary perfusion scintigraphy was performed in a patient with end-stage renal disease due to dyspnea in the post-operative period of kidney transplantation

## Abstract (tr)

Tc-99m-kümelenmiş albüminin (MAA) ekstrapulmoner birikimi, akciğer perfüzyon sintigrafisinde akciğer dışında radyoaktif madde tutulumu olması şeklinde tanımlanır. Eğer Tc-99m-MAA partikülleri venöz kollateraller sayesinde ya da sağdan sola şant nedeniyle akciğere ulaşamadan vücuda yayılırsa veya partiküller akciğer kapillerini tıkayamayacak kadar küçük ise radyoaktif ajan vücudun farklı alanlarında izlenebilir. Tc- 99m-MAA’nın ekstrapulmoner birikimi en sık karaciğerde izlenmekle birlikte, literatürde bazı çalışmalarda beyin, böbrek, tiroid, miyokard, dalak ve vertebralarda da gösterilmiştir. Burada, Tc-99m-MAA’nın beklenmedik hepatik birikiminin tespit edildiği akciğer perfüzyon sintigrafisi görüntülerini sunuyoruz. Bu akciğer perfüzyon sintigrafisi, son dönem böbrek yetmezliği bulunan bir hastaya böbrek transplantasyonu operasyonu sonrası döneminde gelişen nefes darlığı nedeniyle uygulanmıştır.

## Figures and Tables

**Figure 1:**
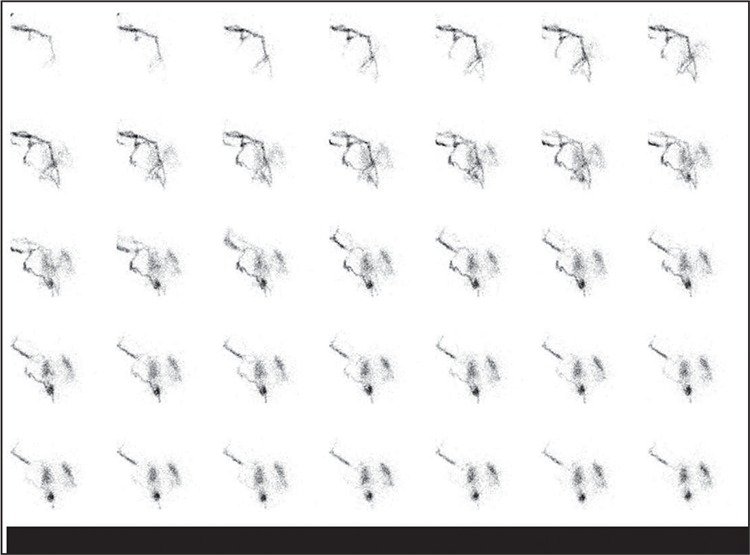
A 25 year-old female patient has been undergoing hemodialysis therapy for 11 years. During hemodialysis period, her arteriovenous fistulas have occluded and aneurysms have developed many times in different vascular locations. Thus, her vascular access was altered to a central venous catheter in 2015. Various venous sites such as bilateral jugular, subclavian and femoral veins had been used for blood exchange due to repetitive venous stenoses. After failure of central venous catheters, she has eventually undergone cadaveric kidney transplantation on May in 2017. She developed dyspnea in the post-operative period. The graft was functioning very well. She was referred to our department for pulmonary perfusion scintigraphy with suspicion of pulmonary embolism. A two head gamma camera (Siemens Symbia T16 SPECT/CT, Germany) was used for scintigraphy, with 80 Mbq of MAA administered intravenously for perfusion imaging. In static and SPECT/CT images, we detected an area that uptakes macroaggregated albumin (MAA) that corresponded to segment 4B in the liver in addition to three filling defects in the lung. After this finding, we acquired dynamic images that focused on the right chest and axillar region, and determined collateral circulation from the axillary region to the liver via lateral chest wall veins. When we injected the radioactive agent into the cephalic vein, an amount of radiopharmeutical was taken by liver via collaterals and caused an uptake in liver parenchyma. Since the superior vena cava (SVC) was not totally occluded, rest of the radioactive agent taken by lungs. There was no uptake in other organs since any connection to the systemic arterial circulation was lacking.

**Figure 2:**
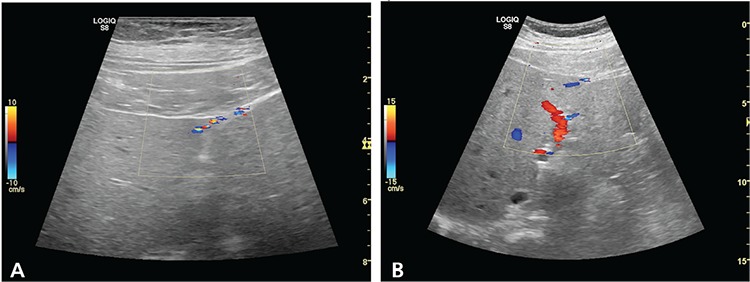
A venography could not be performed due to the risks of nephrotoxicity and embolus. Therefore, we planned for a color doppler ultrasound (CDUS) study. In CDUS, an unusual venous structure that perforated the capsule and entered to the liver parenchyma from segment 4 has been identified (A). Any other pathologic sign could not be seen in the liver parenchyma, the flow direction of that vein was towards the liver, which excluded any liver pathology such as portal hypertension or cirrhosis (B). These findings suggest a collateral circulation via the lateral thoracic veins between the right upper extremity and the liver. When SVC is obstructed, collateral pathways can emerge in the internal mammary, the azygos, the lateral thoracic and the vertebral venous pathways. In addition to SVC obstruction, presence of collateral circulation has been shown following inferior vena cava (IVC) occlusions. A caval-portal shunt is provided by the inferior mesenteric vein, umbilical vein and left renal vein. Intrahepatic collateral veins between proximal and distal segments of the obstruction is also specific to IVC obstructions (1). Extra-pulmonary accumulation of Tc-99m-MAA can be detected if the agent is shunted to the liver directly via venous–venous collaterals before reaching the right atrium, due to right-to-left shunt in the heart or lung and when the particles are degraded into sub-micron sizes. It has been reported that extra-pulmonary accumulation of Tc-99m-MAA was less than 4% among 378 lung scan patients (2). Extra-pulmonary accumulation of Tc-99m-MAA can be detected mostly in the liver as well as the brain, kidney, thyroid, myocardium, spleen and vertebra in several studies (3,4,5,6).
